# Cleaning and Bleaching Impression Wax

**Published:** 1872-06

**Authors:** 


*Cleaning and Bleaching Impression Wax.*—In the "Dental Advertiser" the following method of cleaning wax is given:

"Wax or wax compounds, used in taking impressions, can be made clean and free from all foreign substances by melting in a shallow earthen dish, with one half ounce sulphuric acid to a quart of water. Let it boil fifteen minutes and stir well. Set aside to cool and settle; when the wax has become perfectly hard remove from the dish. All dirt and impurities will be found on the bottom of the cake; cut the settlings from the wax with a sharp knife, after which wash in clean water to remove all traces of acid, and cast into cakes for future use. This method we have tried and know it will clean wax perfectly.

"Wax can be bleached chemically and at the same time cleansed by the following method: Melt the wax and add for each pound two ounces of nitrate of soda, and one ounce of sulphuric acid diluted with nine parts of water. The latter should be added very slowly, while the melted wax is constantly stirred with a glass rod. It is then cooled and set aside after filling the vessel with boiling water. Washing the wax with boiling water until no trace of acid remains, completes the process."

